# Chen-Style Tai Chi for Individuals (Aged 50 Years Old or Above) with Chronic Non-Specific Low Back Pain: A Randomized Controlled Trial

**DOI:** 10.3390/ijerph16030517

**Published:** 2019-02-12

**Authors:** Jing Liu, Albert Yeung, Tao Xiao, Xiaopei Tian, Zhaowei Kong, Liye Zou, Xueqiang Wang

**Affiliations:** 1 Department of Martial Arts, Shanghai University of Sport, Shanghai 200438, China; 2 Depression Clinical and Research Program, Massachusetts General Hospital, Harvard Medical School, Boston, MA 02114, USA; ayeung@mgh.harvard.edu; 3 College of Mathematics and Statistics, Shenzhen University, Shenzhen 518060, China; taoxiao@szu.edu.cn; 4 Department of Physical Education, Qiannan Normal University for Nationalities, Guizhou 558000, China; tianxiaopei1986@163.com; 5 Faculty of Education, University of Macau, Macao, China; zwkong@um.edu.mo; 6 Lifestyle (Mind-Body Movement) Research Center, College of Sports Science, Shenzhen University, Shenzhen 518060, China; 7 Department of Sport Rehabilitation, Shanghai University of Sport, Shanghai 200438, China; qiang897@163.com

**Keywords:** low back pain, VAS, proprioception, joint repositioning sense, Tai Chi, Core Stabilization training

## Abstract

Tai Chi (TC) can be considered safe and effective intervention to improve pain and pain-related functional disability. However, it is unclear that whether aging individuals with Chronic Non-Specific Low Back Pain (CNS-LBP) can achieve positive results. This study, therefore, attempted to explore the effects of TC on pain and functional disability in CNS-LBP patients aged 50 years old or above. Forty-three individuals (aged 50 years old or above) with CNS-LBP were randomly assigned into three groups: Chen-Style TC group (*n* = 15), Core Stabilization training (CST) group (*n* = 15), and control group (*n* = 13). Participants in the TC group participated in Chen-style TC training program (three 60-min sessions per week for 12 weeks), individuals in CST group received 12-week Core Stabilization exercise on the Swiss ball, whereas individuals in the control group maintained their unaltered lifestyle. Pain intensity as primary outcome was measured using the Visual Analogue Scale (VAS), A BiodexSystem 3 isokinetic dynamometer was used to measure knee and ankle joint position sense (JPS) as secondary outcomes at baseline and after the 12-week intervention. TC and CST have significant effects in VAS for CNS-LBP patients (*p*< 0.01, TC group OR CST group versus control group in mean of the post-minus-pre assessment). However, the feature of joint position sense (JPS) of ankle inversion, ankle eversion and knee flexion did not occur, it showed no significant effects with TC and CST. TC was found to reduce pain, but not improve lower limb proprioception in patients with CNS-LBP. Future research with larger sample sizes will be needed to achieve more definitive findings on the effects of TC on both pain and lower limb proprioception in this population.

## 1. Introduction

Chronic non-specific low back pain (CNS-LBP) often occurs in aging populations, which accounts for about 85% of low back pain [1]. Globally, the number of individuals with low back pain has reached epidemic levels, with the estimated mean point prevalence rate of 11.9% and one-month prevalence of 23.2% [2]. Data from the US Burden of Disease Collaborators indicated that low back pain has become the foremost leading cause of disability in the United States [3]. Furthermore, the fiscal impact of low back and neck pain is estimated to be $87.6 billion, which is the third highest proportion of the health care expenditures [4]. Because the pathoanatomical cause of CNS-LBP remains unknown, no specific evidence-based method has been recommended for clinicians to cure CNS-LBP [5]. The clinical practice guidelines suggest that management should focus on reducing pain and its pain-related functional disability [6]. For individuals with CNS-LBP, long-term use of analgesic medicines was associated with psychological distress like depression [7] and increased risk for other health issues such as falls, fractures, and sexual dysfunction [8]. Under this condition, non-pharmacological interventions have received great attention from health professionals [8].

Non-pharmacological methods, such as manual therapy, acupuncture, cognitive behavioral therapy (CBT), massage, and exercise, have been gradually accepted by clinicians to treat CNS-LBP [6]. However, it must be acknowledged that some treatments like CBT are costly and time-consuming, which may not always be readily available for those in both developing and economically disadvantaged countries since they are not covered by health insurance [9]. This makes room for exercise therapy in the management of CNS-LBP. Previous studies have focused on the therapeutic effects of physical exercises for CNS-LBP, including coordination exercise, core stabilization exercise, strength/resistance exercise [10,11,12]. In particular, core stabilization exercise has received greater attention in the management of CNS-LBP [13,14]. Its emphasis is to maximize dynamic spinal stability through increasing trunk muscle strength and endurance (e.g., transversus abdominal, lumbar multifidi, and erector spinae) and optimizing the coordinated contraction of these spinal muscles. When compared to these Western exercise therapies, Eastern traditional mind-body exercises like Tai Chi [15,16,17] and Qigong (Baduanjin and Wuqinxi) [18,19,20] are more enjoyable and preferable exercise modalities to Chinese community-dwelling older persons [21].

Tai Chi (TC) was developed in China as a very effective health-promoting exercise [22,23], and it is widely practiced by people from different age groups in the world [24]. As with physical exercise that focus on muscular strength, cardiorespiratory function, and functional flexibility, multimodal TC training has emphasized mind–body integration; slow-flowing movements should be coordinated with musculoskeletal relaxation, breathing control, and mental concentration in a state of meditation [25,26].

Furthermore, performing TC movements demands neutralization/stabilization of trunk muscles in order to maintain center of gravity regardless of single- and double-leg support, which embody the core stabilization exercise principles [27]. Presumably, it is suitable for old-age persons with low back pain. Two randomized controlled trials investigated the effects of TC in treatment of low back pain [28,29], one of them specifically focused on individuals with CNS-LBP [29]. Results from both studies indicated that TC can be considered safe and effective intervention to improve pain and pain-related functional disability. It needs to be pointed out that researchers in the previous studies recruited middle-aged adults (including retired athletes). It remains unclear for to CNS-LBP patients aged 50 years old or above. Those who are facing decline in physical functions, such as lower-limb proprioception [30], can achieve positive results. Thus, we conducted a randomized controlled trial to investigate the effectiveness of TC for aging individuals with CNS-LBP.

## 2. Method

### 2.1. Experimental Design

This study was a three-armed, randomized controlled design to examine the effects of TC versus core stabilization exercise in treating CNS-LBP as compared to the control group. Study protocol was approved by the ethic committee of the Shanghai University of Sports. Before initiating this study, the trial was registered at the Chinese Clinical Trial Registry (Registration number: ChiCTR-TRC-12002244). Procedures (Years 2012 to 2013) were carried out in accordance with the ethical standards of the Helsinki Declaration. All eligible participants had signed the informed consent forms before the beginning of this study.

### 2.2. Participant Recruitment and Randomization

**Figure 1:**
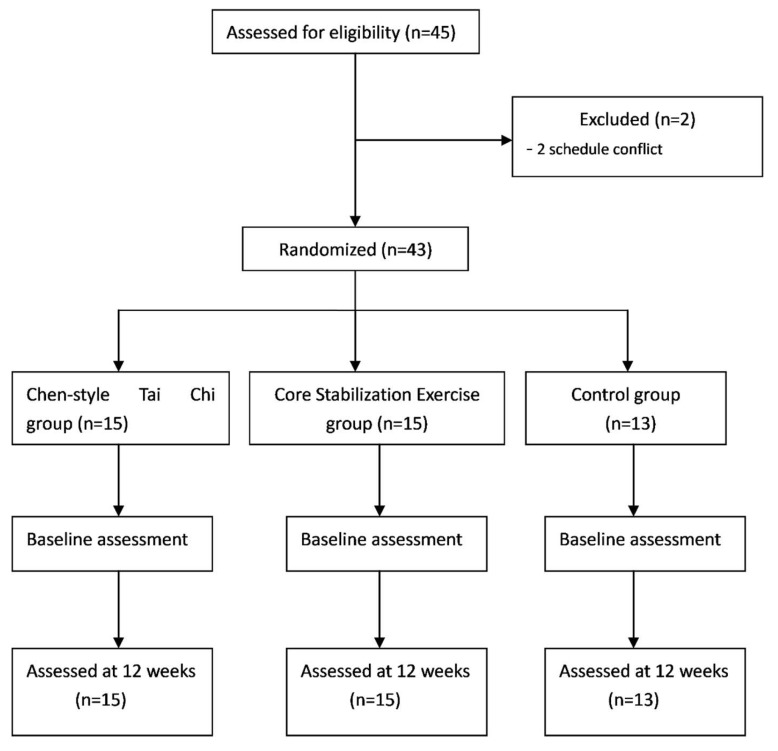
Flow diagram of eligibility assessment, exclusion, inclusion, and analysis.

Study participants were recruited from both the Orthopedic Rehabilitation Center of the Shanghai University of Sports and the Yangpu community. Patients were considered eligible if they met the following criteria: (1) adults aged 50 or above; (2) being diagnosed with CNS-LBP for a minimum of three months; and (3) having the capability to independently ambulate and participate in TC training. Exclusion criteria included: (1) low back pain caused by tumor, rheumatoid arthritis, or infection; (2) the scores ≥8 of the Visual Analog Scale; (3) history of psychiatric disorder and cerebrovascular diseases, neurological disorders, or musculoskeletal disorders; and (4) TC training in the past three months. Basedon the Random Number Generator (https://www.random.org/sequences/), all eligible patients with CNS-LBP were randomly assigned into three groups: Chen-Style TC, active control with Core Stabilization exercise, and non-active control with no intervention. More specifically, 43 eligible participants were first arranged in excel from top (1) to bottom (43). Second, in the Random Sequence Generator, we set up both the smallest value (1) and largest value (43), while three columns (Column 1 = Group 1, Column 2 = Group 2, and Column 3 = Group 3) were predetermined. Third, once we clicked the“get sequence” button, three columns of numbers were generated. Table 1 shows the demographic characteristics of the study participants. The flowchart presents the process of participant selection and experimental implementation (Figure 1).

### 2.3. Intervention Protocol

#### 2.3.1. Chen-Style Tai Chi

The first author who taught TC for more than 30 years had selected 16 Chen-style TC movements [9]: (1) Commencing Form; (2) Buddha’s Warrior Attendant Pounds Mortar; (3) Tuck in Robes; (4) Single Whip; (5) Wave Hands Like Clouds; (6) Double Push Palms; (7) Step Back and Whirl Arms on Both Sides; (8) White Crane Spreads Wings; (9) Diagonal line spread step; (10) Deflect through The Back; (11) the Chopping Hand; (12) Hide Hand and Strike Fist; (13) Six Seals and Four Closings; (14) Single Whip (same as (4)) and Body Defending Punches; (15) Turn-back and Buddha’s Warrior Attendant Pounds Mortar; and (16) Closing Form for CNS-LBP. As mentioned previously, TC movements, in general, involve the rotation of the waist/pelvic region that is like the turning of a wheel on an axle.

Chen-style TC has greater emphasis on silk reeling-spiral movements, alternating fast/slow motion and bursts of internal power, which may provide additional stimulation for lumbar muscles, such as increasing the physical flexibility of joints and muscular strength of low back. TC training lasted for 12 weeks, three times per week with each session of 60 min. There were three phases within 12-week intervention: (1) TC standing posture and individual movement practice in the first stage (four weeks); (2) individual movement training and combination in weeks 5 to 8; and (3) entire routine practice in weeks 9 to 12.

#### 2.3.2. Core Stabilization Exercise and Non-Active Control

Patients with CNS-LBP in the active control group also received 12-week intervention and were instructed with the core stabilization exercise (CSE) on the Swiss ball that had an emphasis on strengthening deep muscles of the abdomen. The CSE routine consisted of six movements (Glute Bridge Pose, Single Leg Bridge, Bridge and Double Knee Flex, Single Leg Bridge and Double Knee Flex, Reverse Bridge, Reverse Bridge and Hip and Knee Flex) [31], and entire training was administered by a certified physical therapist. Training frequency involved three times per week, with 60 min per session. There were two phases throughout this intervention period: (1) learning individual movements in the first four weeks; and (2) individual movement training in a repetitive manner. Patients in the non-active control group throughout this intervention period did not undergo any rehabilitation program while they were asked to maintain unaltered lifestyles.

### 2.4. Outcome Measures

#### 2.4.1. Visual Analogue Scale (VAS) Test

Baseline (before randomization assignment) and post-intervention assessment for all outcome measures were administered by physicians at the Orthopedic Rehabilitation Center who were blinded to group assignment. Pain intensity was considered as a primary outcome in the present study, and it was measured using the Visual Analogue Scale (VAS) [32]. Each patient was asked to mark the location on the 10-cm line that corresponds to the intensity of pain he or she experienced; higher scores indicate greater levels of pain.

#### 2.4.2. Active Position Sense Test

The first section assessed subjects’ joint position matching ability of the knee and ankle in different degrees. A BiodexSystem 3 isokinetic dynamometer (Biodex Medical Systems, Shirley, NY, USA) was used to measure knee and ankle joint position sense (JPS). (1) *Knee Joint Position Test.* Firstly, participants were asked to lay supine on the chair, the left leg is attached to the power arm, and the shoulder, the waist and the thigh are fixed to the seat. Subjects kept their eyes closed and wore headphones with music playing to eliminate visual and auditory stimuli from the testing apparatus. The knee joint was passively set to 45-degree flexion from 90-degree flexion, and the position was held for 10 s to allow each participant to perceive where his or her tested leg is located and then the tested leg was extended by the assessor. This passive flexion was repeated three times. Secondly, the active test was performed after having the knee passively placed in the beginning position (90-degree flexion), the subject was asked to move the knee actively back to the test position. The subject was again asked to push on the stop button when he or she thought the test position was reached. This was repeated three times. (2) *Ankle Joint Position Test.* Each subject was positioned semi-recumbent on the associated special testing chair, with the calf of the tested leg resting on a 40-cm high platform. The hip and knee were positioned at a 45° flexion, and the talocrural joint was in neutral position. The bare foot of the subject was aligned with the axis of the dynamometer and attached to the footplate by a very small wrap to reduce cutaneous receptor input. Subjects kept their eyes closed and wore headphones with music playing to eliminate visual and auditory stimuli from the testing apparatus. There were two reference degrees: ① ankle at 10° inversion and ② ankle at 10° eversion. The subject’s foot was first passively moved by the investigator to the maximal inversion or eversion position. The investigator then moved the foot to the two reference positions. This test position was maintained for 10 s, with each subject instructed to concentrate on the position of the foot. The foot was then passively brought to maximal inversion or eversion; this passive inversion or eversion was repeated three times. Secondly, the active test was performed except after having the foot passively placed in the test position and moved to maximal eversion, the subject was asked to move the foot actively back toward eversion or the inversion test position with a speed of 5°/s. The subject was again asked to push the stop button when he or she thought the test position was reached. This trial was repeated three times. The error with which the subject reproduced the initial position was subsequently calculated. Average scores of the three trials were used for analysis, and the average value was termed the absolute angle error. The absolute error is the difference in absolute value in degrees between the position chosen by the subject and the test-position angle.

### 2.5. Statistical Analysis

The difference values between the baseline (before randomization assignment) and post-intervention assessments for all four of the features in our interests, from categories of proprioception, VAS, were calculated and analyzed, in order to see if the means of the post-minus-pre differences are different among groups. Specifically, for each of the four features, we conducted a regression analysis using as the dependent value the difference value obtained by subtracting pre-intervention measure from post-intervention measure for each of the 43 patients from three groups; the independent values include the two indicator variables GROUP_TC and GROUP_CR for grouping information (i.e., GROUP_TC = 1 stands for the Tai Chi group and GROUP_CR = 1 stands for the Core Stabilization group), an indicator variable GENDER (GENDER = 0 stands for female and GENDER = 1 stands for male), HEIGHT (in CM) and WEIGHT (in KG). Therefore, the post-pre difference value of the *j*th feature of the *i*th subject, $d_{ji}$, is modeled with a multiple regression as

(1) $$d_{ji}=\beta_{j0}+\beta_{j1}GROUP_{TC}+\beta_{j2}GROUP_{CR}+\beta_{j3}GENDER+\beta_{j4}HEIGHT+\beta_{j5}WEIGHT+\varepsilon_{ji}$$

where $\varepsilon_{ji}$ is normally distributed with mean.

We focus and report significant associations between grouping and each *j* of the 52 features, with significance level pre-set as 0.05. For each *j*, we tested two null hypotheses $\beta_{j1}=0$ and $\beta_{j2}=0$. If the first null hypothesis is rejected at level 0.05, we would conclude based on our data that the mean post-minus-pre difference for the *j*th feature is not equal between the TC group and the control group; if the second null hypothesis is rejected at level 0.05, we would conclude based on our data that the mean post-minus-pre difference for the *j*th feature is not equal between the Core Stabilization group and the control group. In the following two tables, we respectively list the feature names for which the above-stated group comparisons are significant (we also list the corresponding *p*-values on the side).

## 3. Results

A total of 45 eligible patients with CNS-LBP were recruited and then they were screened against the eligibility criteria. In the screening, two participants were excluded because they could not participate due to scheduling conflicts. Thus, 43 participants were finally included in this study. No significant difference for demographic information was observed among the TC, CS and control groups (Table 1). There is no significance among the three groups.

From Table 2 below, we can see that the feature of VAS occurs in both groups, suggesting that Tai Chi and Core Stabilization training have significant effects on VAS for CNS-LBP patients. However, the features of joint position sense (JPS) of ankle inversion, ankle eversion and knee flexion did not occur; it showed no significant effects with Tai Chi and Core Stabilization training.

## 4. Discussion

The results of this study showed that, for patients with CNS-LBP, both Tai Chi and Core Stabilization exercises had positive effects on pain, but not on lower limb proprioception. The positive finding of Tai Chi on pain is of particular interest as this was the primary hypothesis tested and the main objective of this study. The mechanisms of Tai Chi’s effect on lower back pain are not fully understood. They might include general effects due to exercise such as increased flexibility and mobility of structures; improved muscle strength and endurance; increased tensile strength of ligaments and capsules; increased cardiopulmonary function, reduced stress, anxiety, and depression; and changes in health beliefs and health-related locus of control [33].

It is encouraging that we were able to show positive effects of Tai Chi on pain with a relatively small sample size in this study. Findings from previous studies on the effect of Tai Chi and other active exercises on pain were mixed; some studies reported positive outcomes [33], while others did not [34]. Nevertheless, active exercises including Tai Chi, Pilates, and yoga are the first line of treatment recommended by many well-known treatment guidelines of CNS-LBP, while rest, passive exercises and meditation are not [35]. The varied results of Tai Chi on lower back pain around patients with CNS-LBP may be due to a range of factors: the different characteristics of the studied patient populations (e.g., underlying pathologies, age range, comorbid medical and psychiatric conditions, the level of pain severity, the formats of interventions (the style, duration, and intensity of Tai Chi training), the design of the study (e.g., the sample size, and the characteristics of controls used and the interventions provided to the controls etc.)). Similar to the literature of Tai Chi on lower back pain, the number of studies on the effects of Tai Chi on improving lower limb proprioception is growing. Overall results of a recent meta-analysis [30] indicated positive outcomes of Tai Chi on lower limb proprioception. On the other hand, a number of individual studies did not show significant positive outcomes of Tai Chi on lower limb proprioception [36,37,38]. Similar factors described for the varied effects of Tai Chi on pain may also influence the outcomes of Tai Chi on lower limb proprioception.

The outcomes of this study have contributed to the relatively small amount of literature on the effects of Tai Chi on pain among patients with CNS-LBP. There are a number of strengths in this study, including the use of randomized controls, experienced Tai Chi instructor and certified physical therapist in providing interventions, blind assessors, and the use of accepted methods for outcome measurements including VAS for pain and isokinetic dynamometer for proprioception measurement. There are several limitations in this study: firstly, the sample size was relatively small, which may have contributed to the negative outcomes in proprioception. Secondly, participants in this study were not blind to intervention allocation, which is hard to achieve in active exercise research including the use of Tai Chi. The results might have been confounded by positive anticipation of outcomes by participants in the intervention group. Thirdly, information on comorbid medical and psychiatric conditions was obtained by patients’ self-reporting, which could be biased by under-recognition of psychiatric disorders due to the lack of systematic depression screening and diagnostic interviewing processes. Fourthly, psychological characteristics like pain catastrophization, which had been reported as potential moderators of pain, were not ascertained in this study. Lastly, as the studied participants were homogeneous Chinese populations in Shanghai, the results may not be generalizable to patients in other areas in China or to non-Chinese populations.

The effects of clinical treatments on CNS-LBP have been varied. It has been argued that CNS-LBP is a heterogeneous group of clinical conditions and subsequently their treatment outcomes were different. Future directions include better understanding of patients with chronic, non-specific back pain and examine the possibility of refining their classification using advanced diagnostic technologies and biomarkers. Similarly, the study on the effects of Tai Chi as an active exercise for treating CNS-LBP would benefit from a more standardized treatment protocol including the type of Tai Chi taught, the duration and the frequency of training, as well as the specific training provided in each class.

## 5. Conclusions

Tai Chi was found to reduce pain, but not improve lower limb proprioception in patients with CNS-LBP. Future research with larger sample sizes will be needed to achieve more definitive findings on the effects of Tai Chi on both pain and lower limb proprioception in this population.

## Figures and Tables

**Table 1:** Demographic characteristics of all subjects in Tai Chi, Core Stabilization and Control groups.

Variable	Tai Chi (*n* = 15)	Core Stabilization (*n* = 15)	Control (*n* = 13)
Female (%)	73.3	73.3	76.9
Age(y)	58.13 ± 5.38	58.4 ± 5.08	60.67 ± 2.58
Body weight	58.93 ± 9.93	63.33 ± 9.08	63.47 ± 12.05
Height	159.53 ± 7.24	162.53 ± 8.21	159.00 ± 7.17

Note: Chi-square test was used for Female (%); one-Way ANOVA was used for the other three indexes among three groups.

**Table 2:** Visual Analogue Scale (VAS), joint position sense (JPS) of ankle inversion, ankle eversion and knee flexion at baseline and 12 weeks in Tai Chi, Core Stabilization and Control groups.

Parameters	Tai Chi		Core Stabilization		Control	
	Pre (*t* = 0)	Post (*t* = 12 W)	Pre (*t* = 0)	Post (*t* = 12 W)	Pre (*t* = 0)	Post (*t* = 12 W)
VAS	5.67 ± 0.81	3.47 ± 0.99 **	5.67 ± 0.72	4.27 ± 0.79 ^△△^	5.85 ± 0.89	5.85 ± 0.8
JPS of ankle inversion	9.72 ± 4.88	2.35 ± 2.05	10.24 ± 7.58	6.18 ± 4.46	7.37 ± 4.94	5.98 ± 3.47
JPS of ankle eversion	5.14 ± 3.17	2.72 ± 1.88	6.08 ± 3.39	4.59 ± 2.53	4.21 ± 3.68	6.64 ± 4.51
JPS of knee flexion	9.98 ± 6.49	0.81 ± 0.5	9.44 ± 8.69	6.46 ± 5.72	7.12 ± 2.76	5.86 ± 4.51

Note: ** = Tai Chi group versus control group in mean of the post-minus-pre assessment at the threshold of *p* < 0.01; ^△△^ = Core Stabilization group versus control group in mean of the post-minus-pre assessment at the threshold of *p* < 0.01.
